# Chemical analysis and computed tomography of metallic inclusions in Roman glass to unveil ancient coloring methods

**DOI:** 10.1038/s41598-021-90541-8

**Published:** 2021-05-27

**Authors:** Francesca Di Turo, Giulia Moro, Alessia Artesani, Fauzia Albertin, Matteo Bettuzzi, Davide Cristofori, Ligia Maria Moretto, Arianna Traviglia

**Affiliations:** 1 Center for Cultural Heritage Technology (CCHT), Istituto Italiano di Tecnologia, 30175 Venice, Italy; 2 Department of Molecular Science and Nanosystems, Ca’ Foscari University of Venice, 30172 Venice, Italy; 3 Historical Museum of Physics and the Enrico Fermi Study and Research Center - CREF, 00184 Rome, Italy; 4 Department of Physics and Astronomy, University of Bologna, 40127 Bologna, Italy; 5 INFN - National Institute of Nuclear Physics, 40127 Bologna, Italy; 6 Ca’ Foscari University of Venice, Centre for Electron Microscopy “Giovanni Stevanato”, 30172 Venice, Italy

**Keywords:** Glasses, Colloids, Corrosion

## Abstract

This paper describes the analysis of two near-spherical metallic inclusions partially incorporated within two Roman raw glass slags in order to elucidate the process that induced their formation and to determine whether their presence was related to ancient glass colouring processes. The theory of metallic scraps or powder being used in Roman times for glass-making and colouring purposes is widely accepted by the archaeological scientific community, although the assumption has been mainly based on oral traditions and documented medieval practices of glass processing. The analysis of the two inclusions, carried out by X-ray computed tomography, electrochemical analyses, and scanning electron microscopy, revealed their material composition, corrosion and internal structure. Results indicate that the two metallic bodies originated when, during the melting phase of glass, metal scraps were added to colour the material: the colloidal metal–glass system reached then a supersaturation condition and the latter ultimately induced metal expulsion and agglomeration. According to the authors’ knowledge, these two inclusions represent the first documented and studied finds directly associated with the ancient practise of adding metallic agents to colour glass, and their analysis provides clear insights into the use of metallic waste in the glass colouring process.

## Introduction

**Figure 1:**
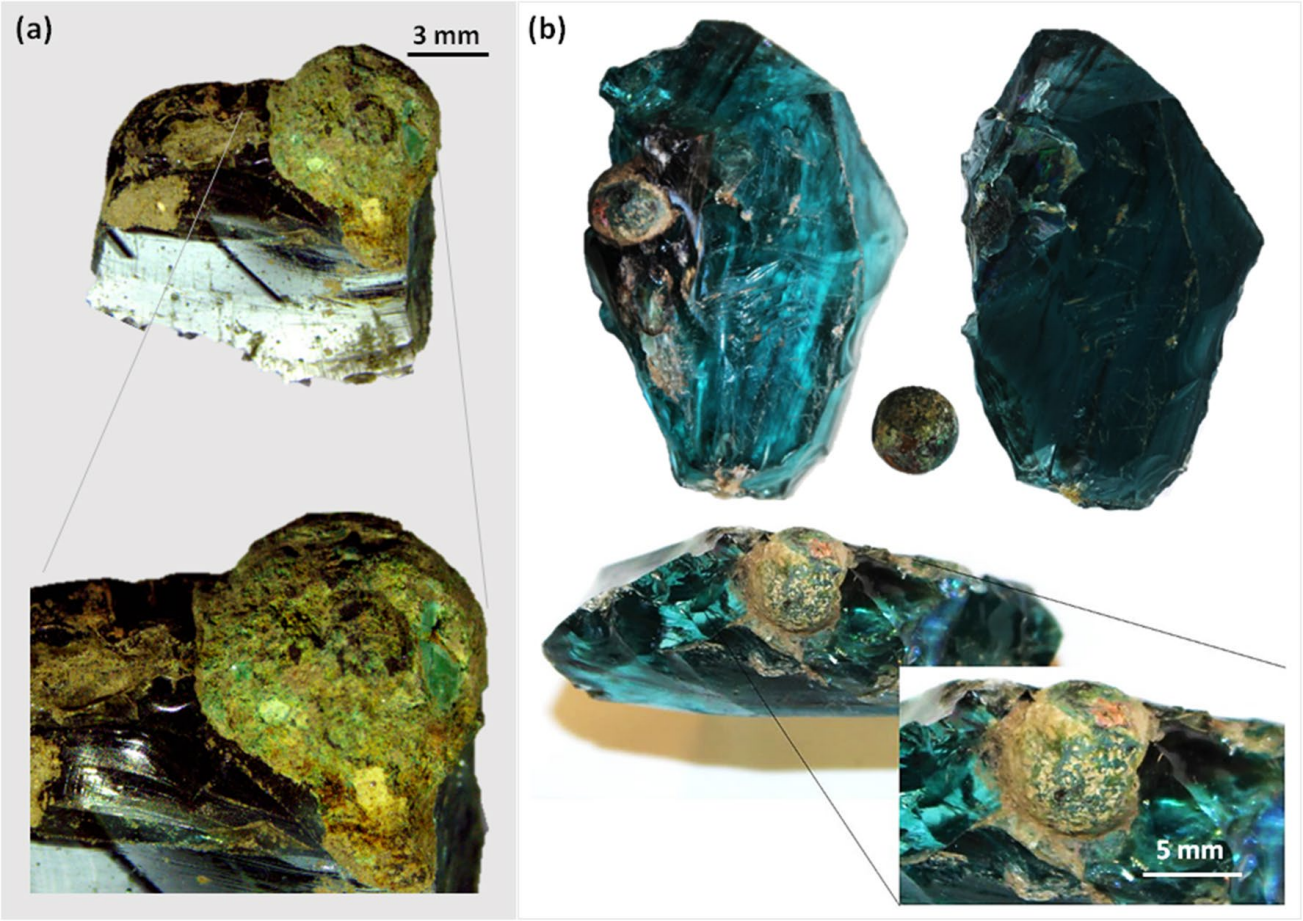
Metallic near-spherical inclusions partially embedded in glass: (**a**) inclusion #1 and (**b**) inclusion #2, before and after extraction. (Image: Giulia Moro).

This paper focuses on the analysis of two metallic near-spherical inclusions that have been found partially incorporated within two chunks of ancient raw glass slags, the nature and function of which could not be initially related to any known phenomenon explicitly described in archaeological literature. The two chunks were part of a remarkable assemblage including glass cullets and other chunks of glass slags (i.e., blobs of raw or recycled glass collected, ground up and recycled to be re-melted and eventually worked into proper objects) that was identified on plough surface during an archaeological prospection on agricultural fields in the outskirts of a major ancient Roman city, Aquileia (Italy). Visual inspection of the glass chunks had revealed that two of them partially incorporated each a small near-spherical metallic inclusion, with diameters of about 6 mm (inclusion #1) and 10 mm (inclusion #2), respectively (Fig. 1a,b). The inclusions were embedded within the glass body and their surface was only partially exposed. Physicochemical analyses of a selection of all the discovered glass slags (that are over 450) had shown that they were mainly made of soda–lime-silica glass, typical of the Roman Imperial period^1,2^, and that they pertained to a period between first and fourth century AD. Their presence in such an elevated number in one single location has led archaeologists to conjecture that in antiquity a Roman glass furnace was likely located in the vicinities. On this basis, the metallic inclusions were interpreted as a waste of the glass-working process. Discussion about glass-making and glass-working’s waste in literature is limited and—at the best of the authors’ knowledge—similar metallic residuals have never been analysed by means of physicochemical analyses.

Coloured glass was obtained in the past by adding minerals or metals—often in form of scraps or powders—to the melted glass, as they act as chromophores^3,4^. The theory of the use of metallic scraps or powder is widely accepted by the archaeological scientific community, although this assumption has been mainly based on oral traditions and documented medieval practice of glass working. This process was carried out in the second stage of glass fusion, when frit blocks of glass, manufactured in so-called primary furnaces, were grounded into chunks, and then re-melted in so-called secondary furnaces^5^. During this second stage, Roman glassmakers used their empirical expertise to select and mix opacifiers, chromophores and modify furnace conditions for tuning the final appearance of glass. Typical examples of opacifiers or chromophores were: lead antimonate and stannate, added to obtain opaque yellow gradations^6^; copper, used for colour shade from turquoise to green; cobalt, utilised to obtain deep-blue; manganese, used for violet shade; and iron oxides, used either for light blue (Fe^2+^) or yellow (Fe^3+^) depending on furnace conditions^7,8^.

This study was thus undertaken to validate the hypothesis of the inclusions being glass-working by-products, to examine how their presence could relate to glass colouring practises and to understand their mechanism of formation. The outcomes of the analyses of their composition and micro-structure elucidated the genesis of the inclusion and their formation process.

## Results

Initial visual inspection showed high roughness and heterogeneous distribution of crusts with various colours on the surfaces of the inclusions: inclusion #1 exhibited green shaded crusts (Fig. 1a), while inclusion #2 showed a more variegate texture with additional orange-yellow and pale grey crusts (Fig. 1b). Their appearance and the occurrence of chromatically different areas suggested the presence of a surface *patina*, a corrosive compounds’ layered structure. Due to conservation practises favouring archaeological object integrity and the need to proceed in the analysis of the items in the least invasive way, the inclusion #1 was analysed while still embedded in the glass chunck, whereas the inclusion #2 was extracted and sectioned by partially removing a thin semi-spherical portion (max. thickness around 2 mm), left attached to the main body by a small edge, in order to expose the inclusion’s core. A description of the sectioning procedure is detailed in ‘Supplementary Materials’.

A step-by-step analytical workflow was designed to investigate the inclusions by progressing from the outside to the inside: this included an initial evaluation from a morphologic-morphometric point of view, followed by the examination of the composition of the external layers and a final step of investigation of the core’s composition (only for inclusion #2).

In the execution of the procedure, X-ray Computed Tomography (CT) was first used to determine the internal structural morphology of the two inclusions, to ascertain material heterogeneities of their bulk, and to determine the thickness of the corroded layers. The elemental composition of the external layers and the corrosion compounds of the *patina* were characterised by means of Field Emission Scanning Electron Microscopy coupled with Energy Dispersive Spectroscopy (FESEM-EDS) and Voltammetry of Immobilized Microparticles (VIMP), respectively. After sectioning it, the internal composition of the inclusion #2 was further investigated with FESEM-EDS.

### Tomographic imaging

X-ray Computed Tomography is a non-invasive imaging technique based on the variations of absorption along the path of the X-ray beam. Since the absorption depends on the atomic number and density of the materials, the technique gives information about their distribution in the investigated object. The acquisition of multiple 2D radiographic images of the item (the so-called projections) over 360 (or 180) degrees and the application of *ad-hoc* tomography reconstruction algorithms result in a set of cross-sectional images of the object (the so-called tomographic slices). In specific cases, additional processing is required to optimize the reconstruction—in this case, the beam-hardening correction. Finally, the use of rendering software re-creates a complete 3D imaging of the investigated object, from the outside morphology to the inner details.

The CT scanning was performed on the inclusion #1 in order to differentiate the object from the surrounding glass and from the *patina* based on their different X-ray absorption, while it was carried out on the semi-spherical cap of the inclusion #2 in order to imaging the material components in the structure.

**Figure 2:**
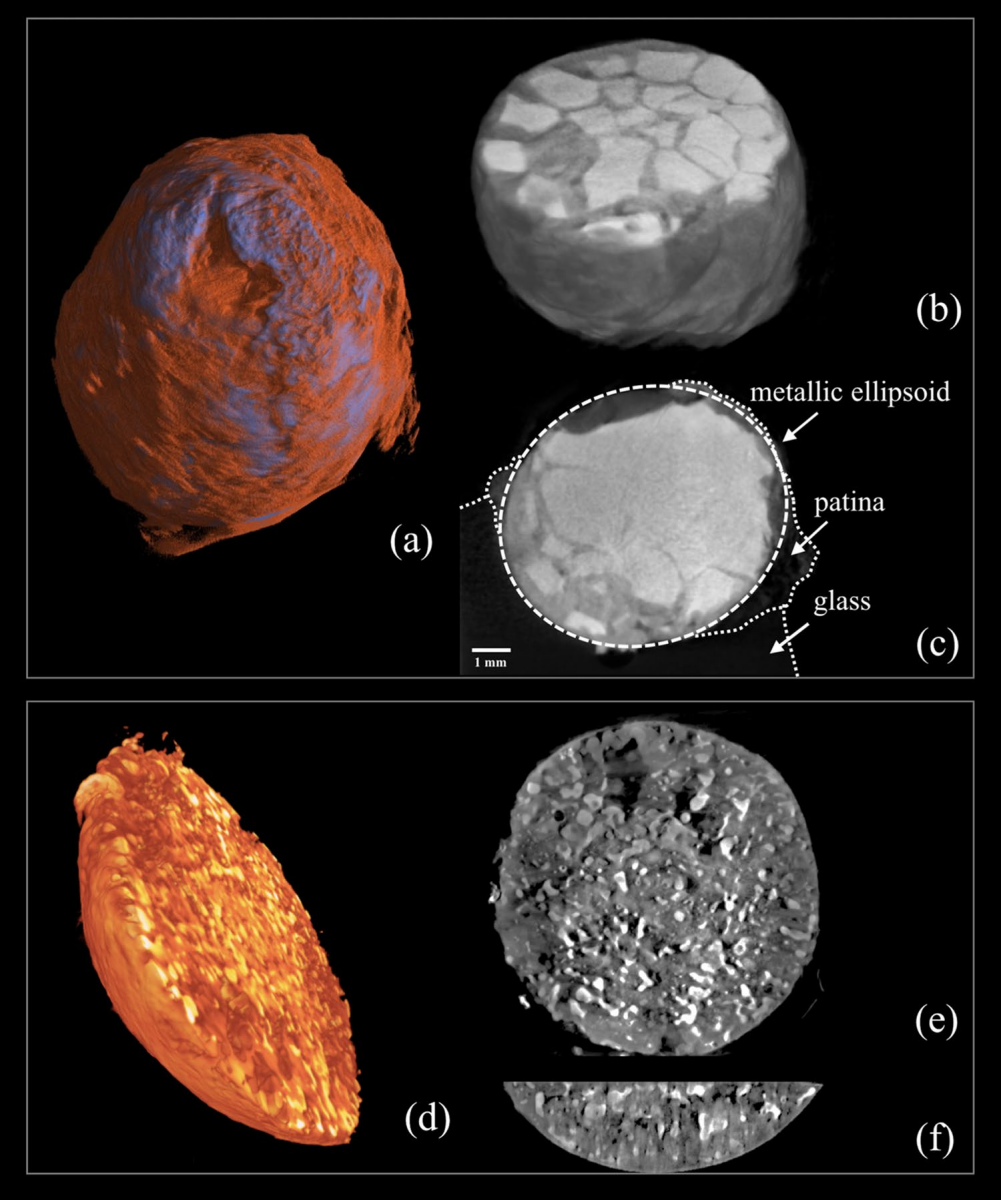
Tomographic investigation of the inclusions. (**a**) The 3D tomographic rendering of inclusion #1 shows the external morphology and distribution of the corrosion patina (in orange); the 3D horizontal (**b**) and vertical (**c**) sections disclose the internal morphology, with the core composed of metal fragments, and the distribution of glass, patina and the metallic ellipsoid. (**d**) The 3D tomographic rendering of the semi-spherical cap of inclusion #2 shows its external morphology; 3D horizontal (**e**) and vertical (**f**) sections show the clustering of the internal materials in small globules and their distribution (Images: Matteo Bettuzzi, Fauzia Albertin).

*Inclusion #1* The obtained 3D reconstruction and volume rendering displayed the external and internal morphology of the inclusion #1 (Fig. 2a–c). Measurements performed by means of tomographic volume and sections showed that the inclusion presented a thick corrosion *patina* on the surface exposed to the environment (in orange in Fig. 2a) with an estimated thickness ranging between 150 and 700 µm, which was absent on the surface of the inclusion still embedded in the glass bulk. This distribution indicates that the corrosion process started after the inclusion #1 was incorporated into the glass chunk, while the glass prevented its formation over part of the surface.

The CT scanning was also used to measure the overall shape of the inclusion, which turned out to be ellipsoidal, and not spherical, with one axis longer than the other by about 1 mm. The 3D reconstruction clarified the inner structure of the object, revealing that the core was not uniform and was instead fragmented and composed of metallic fragments (Fig. 2b,c). The latter were smaller and more easily identifiable in the portion of the metal inclusion still embedded into the glass and the fragments were lodged in a material with less opacity to X-rays (as shown in Fig. 2b,c).

**Figure 3:**
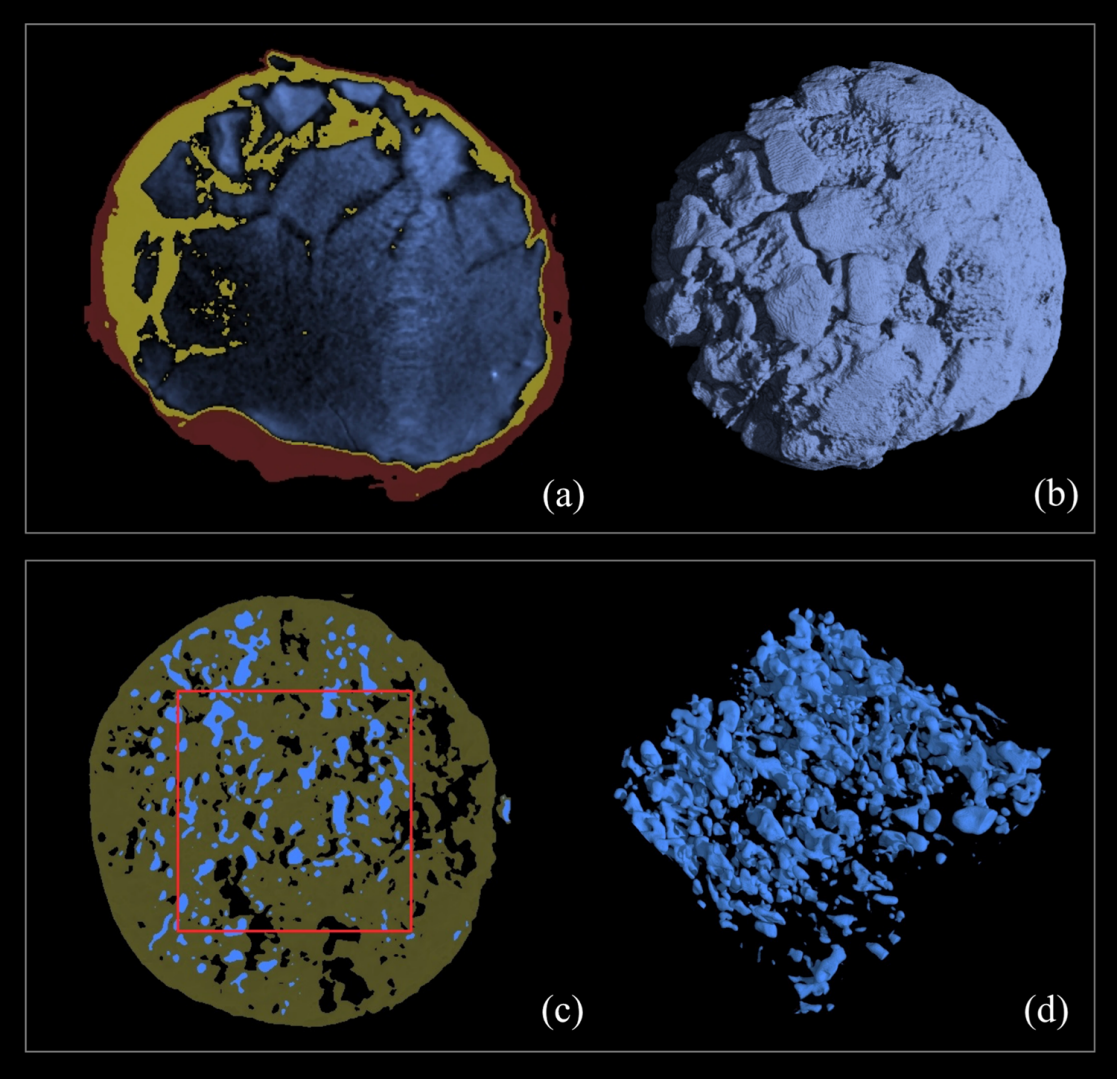
Tomographic volume fractions study of the different materials of inclusions**. **(**a**) and (**b**) the results of the imaging segmentation of Inclusion #1 where the different materials are shown in red—the *patina*, yellow—glass, and blue—metal. (**c**) and (**d**) the results of Inclusion #2 with the different materials presented in black—the empty areas, green—glass, and blue—metal (Images: Matteo Bettuzzi, Fauzia Albertin).

The morphology of the inclusion was also studied from a quantitative point of view. The volume fractions of the different materials were evaluated using the histogram plot analysis and introducing opportune thresholding. The volumetric analysis of Inclusion 1 was performed excluding the glass bulk. The investigation showed that the metallic sphere’s components, measured by volume, can be identified as 18% of corrosion patina, 63% of fragments (metal), and 19% of another material (presumably glass) in which the fragments are lodged. The results of the imaging segmentation are shown in Fig. 3a and b, where the different materials are shown in red, yellow, and blue respectively. *Inclusion #2* The investigation performed on the semi-spherical portion of inclusion #2 revealed a quite different morphology compared to the previous one (Fig. 2d–f). As shown in Fig. 2d, the external *patina* was very limited on it and not appreciable in X-ray imaging. The clustering of internal materials appeared as small globules with a jagged structure. The tomographic investigation revealed a uniform distribution of these globules, from the outer surface to the inner bulk of the object (Fig. 2e,f). The CT scanning also revealed a difference in absorption between the prevailing material and the rest, which appeared almost transparent in the rendering, leading to the hypothesis of an uneven mixture of heavy metal/s and glass.

The beam-hardening correction enabled an accurate analysis of the histogram plots, uncovering tiny differences in the transparent areas and the presence of empty zones in addition to glass. To evaluate their volume fractions, the investigation of the histogram plot was performed on an (internal) sub-volume. The analysis showed that 14% of volume was empty, while 75% was occupied by glass and 11% by metal. The results of the imaging segmentation are shown in Fig. 3c and d, where the different materials presented in black, green, and blue, respectively.

### Material composition

#### External examination (inclusion #1 and #2)

**Figure 4:**
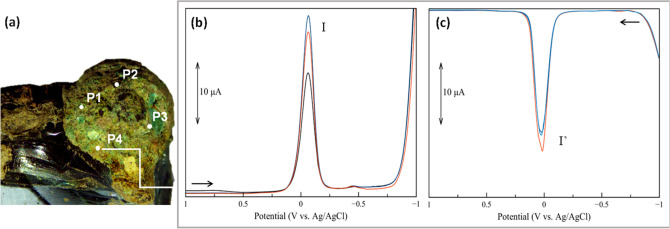
Distribution of the sampling points on the surface of inclusion #1 investigated by VIMP and FESEM-EDS (**a**). Overlap of cathodic (**b**) and anodic (**c**) voltammograms of microparticles (triplicate measurements) collected in P4. (Image: Francesca Di Turo, Giulia Moro).

**Figure 5:**
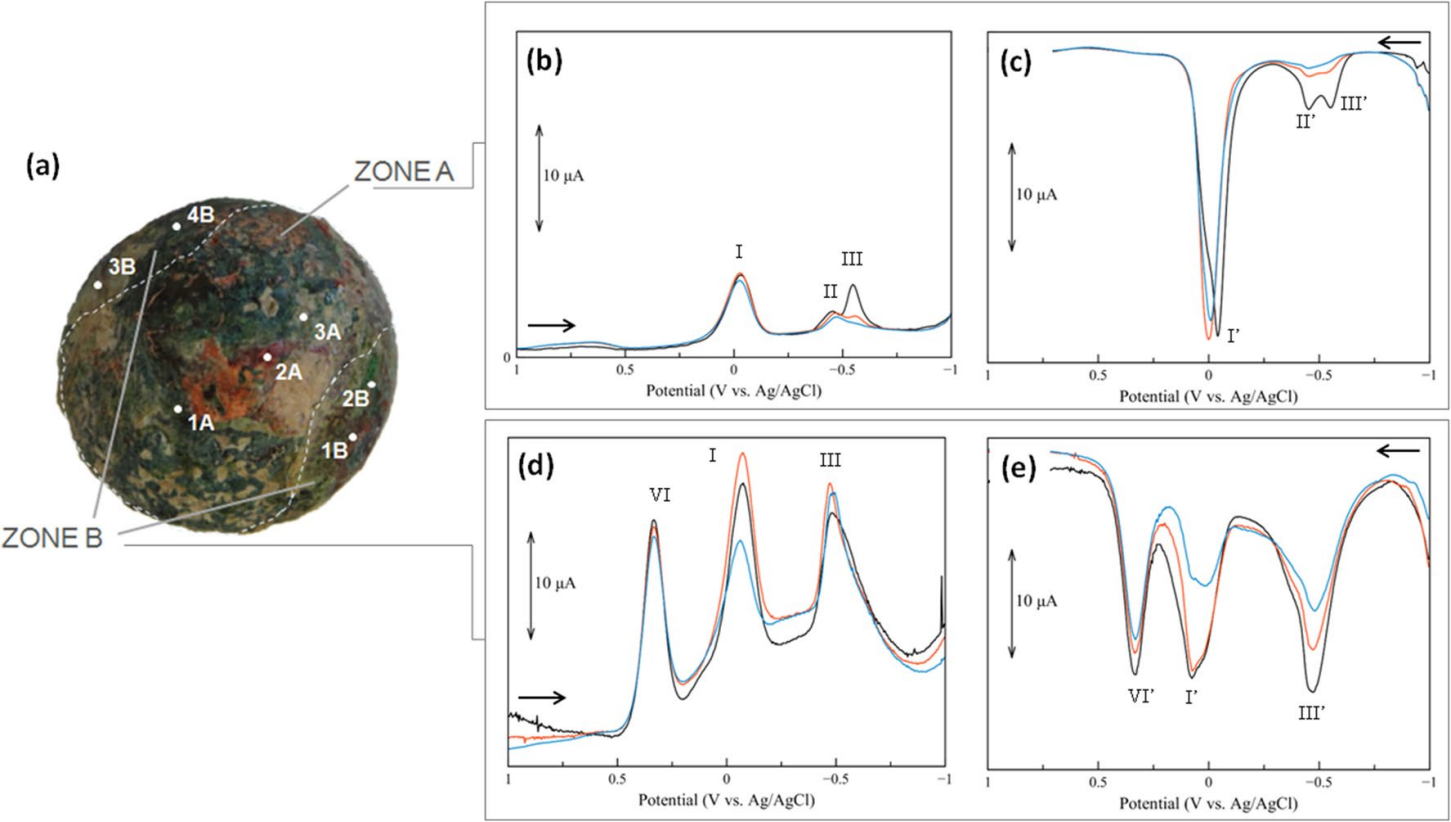
Distribution of the sampling points of Zone A (previously embedded in the glass matrix) and Zone B on inclusion #2, these points were investigated by VIMP and FESEM-EDS (**a**). Overlap of triplicate cathodic (**b**–**d**) and anodic (**c**–**e**) voltammograms of microparticles. The voltammograms representative of Zone A (**b**–**c**) are characterized by an intense peak (I and I’, respectively) in the region 0.00–0.06 V vs Ag/AgCl, while the one Zone B (**d**–**e**) shows multiple processes of comparable intensity (peaks I, II, IV and I’,II’,IV’). (Image: Francesca Di Turo, Giulia Moro).

The composition of the *patina* layers was characterised by means of VIMP and FESEM-EDS in different spots, as depicted in Figs. 4a and 5a.

First used by Scholz et al.^9^, VIMP is an electrochemical technique applied for the characterisation of corrosion products on archaeological *patinas* based on the identification of redox potentials. Here, the analysis of solid microparticles was performed by Square Wave Voltammetry.

FESEM-EDS provides topographical and elemental information (Z > 4) at magnifications far beyond the limitations of light microscopy. In this study, the backscattered electron (BSE) signal was employed to record images where the pixel intensity depends on the local average atomic number (the higher the Z, the brighter the pixel).

*Inclusion #1* Microparticles from four sampling points on the exposed surface showed in Fig. 4a were studied by VIMP. Representative cathodic and anodic voltammograms (Fig. 4b,c, respectively) are characterised by a unique redox process identified by a peak at 0.00 V vs. Ag/AgCl. These signals were ascribed to reduction and oxidation of cuprite (Cu_2_O)^10,11^. It is worth to note that the intensity of the signal depends on the amount of microparticle immobilised on the electrode surface. The presence of copper was confirmed by the percentages of this metal detected by EDS in the range of 52–62 wt%, as showed in Table 1. No other elements and/or corrosion compounds were detected on the surface of the inclusion #1. The analyses suggested that the inclusion was made of copper and that the thick *patina* layer consisted of cuprite.

**Table 1:** Elemental composition of the sampling points of inclusion #1 mapped in Fig. 4a. Data collected with FESEM-EDS.

Element	Z	P1		P2		P3		P4	
		Wt%	W Wt%	Wt%	W Wt%	Wt%	W Wt%	Wt%	W Wt%
Carbon	6	21.1	0.6	20.3	0.6	24.1	0.6	15.3	0.6
Oxygen	8	25.5	0.6	17.8	0.6	21.5	0.6	23.2	0.6
Sodium	11	–	–	–	–	–	–	–	–
Magnesium	12	–	–	–	–	–	–	–	–
Aluminium	13	0.52	0.10	–	–	0.66	0.10	0.10	0.10
Silicon	14	1.20	0.09	–	–	1.55	0.09	1.48	0.09
Phosphorus	15	–	–	–	–	–	–	–	–
Chlorine	17			–	–	0.09	0.01	–	–
Potassium	19	0.08	0.01	–	–	–	–	–	–
Calcium	20	–	–	–	–	–	–	–	–
Iron	26	–	–	–	–	–	–	–	–
Copper	29	51.6	0.6	61.9	0.8	52.1	0.6	59.2	0.7
Tin	50	–	–	–	–	–	–	–	–
Lead	82	–	–	–	–	–	–	–	–
Sum		100.0	2.0	100	2.0	100	2.0	100	2.1

*Inclusion #2* After the extraction from the glass chunk of this inclusion, the face originally exposed to the environment was labelled as Zone A, while the one previously surrounded by the glass was labelled Zone B, as shown in Fig. 5a. The voltammograms of triplicate measurements of microparticles from Zone A (Fig. 5b,c) showed similar reduction and oxidation peaks of copper observed in the inclusion #1. The cathodic peaks of the voltammograms in Fig. 5b were assigned to proton-assisted reduction of cuprite (Cu_2_O, peak I), tenorite (CuO, peak II) and lead oxides (peak III)^12^. The same peaks were identified in the anodic voltammograms, confirming the oxidative dissolution of the metallic deposits generated in the previous cathodic scan (Fig. 5c). VIMP analyses on microparticles from Zone B (Fig. 5d,e) showed a different voltammetric profile. Apart from the signals related to copper and lead oxides (peaks I and III, I’ and III’ in Fig. 5d,e, respectively), an additional signal at 0.30 V vs. Ag/AgCl (peak IV and IV’, Fig. 5d,e, respectively), associated to the presence of other metallic compounds was observed. EDS analysis confirmed that copper and lead were the main elements of the *patina*, with total percentages in the range of 50–70 wt% depending on the sampled area (the elemental composition of each sampling point is reported in Table 2). The presence of tin (maximum of 4.6 wt%) provided additional information about the composition of the original alloy. The elemental composition indicated that inclusion #2 is made of leaded bronze (Cu–Sn–Pb alloy). Other metals found on the *patina* were iron (up to 2%), aluminium (up to 1.7 wt%) and magnesium (up to 0.5 wt%).

**Table 2:** Elemental composition of the sampling points of Zone A and Zone B of inclusion #2 mapped in Fig. 5a. Data collected with FESEM-EDS.

Element	Z	Zone A						Zone B							
		1A		2A		3A		1B		2B		3B		4B	
		Wt%	W Wt%	Wt%	W Wt%	Wt%	W Wt%	Wt%	W Wt%	Wt%	W Wt%	Wt%	W Wt%	Wt%	W Wt%
Carbon	6	21.0	0.6	13.5	0.5	25.2	0.4	24.9	0.8	27.1	0.8	28.0	1.6	19.3	0.6
Oxygen	8	30.2	0.6	13.8	0.5	18.1	0.3	17.9	0.6	25.7	0.7	25.4	0.7	27.2	0.6
Sodium	11	–	–	–	–	–	–	–	–	–	–	0.44	0.09	–	–
Magnesium	12	–	0.09	–	–	0.18	0.04	–	–	–	–	–	–	0.50	0.10
Aluminium	13	0.81	0.08	–	–	1.66	0.04	2.29	0.10	0.71	0.10	0.78	0.07	0.65	0.10
Silicon	14	4.30	0.13	–	–	0.53	0.04	0.56	0.10	5.88	0.18	1.33	0.08	5.73	0.09
Phosphonts	15	1.43	0.11	–	–	–	–	–	–	–	–	3.77	0.14	–	–
Chlorine	17	–	–	–	–	0.56	0.04	0.39	0.10	–	–	0.98	0.12	–	–
Potassium	19	–	–	–	–	–	–	2.56	0.10	–	–	0.61	0.09	–	–
Calcium	2.0	2.90	0.17	–	–	0.71	0.05	1.78	0.10	0.95	0.14	3.54	0.15	–	–
Iron	26	1.91	0.24	–	–	–	–	–	–	–	–	0.89	0.19	–	–
Copper	29	7.8	0.3	–	–	48.8	0.8	37.6	0.7	35.8	0.8	–	–	43.1	0.6
Tin	50	4.60	0.12	–	–	1.50	0.12	2.80	0.10	–	–	–	–	–	–
Lead	82	24.3	0.8	72.7	0.6	2.86	0.14	9.37	0.10	3.5	0.4	34.3	0.9	3.3	0.5
Sum		99.0	3.0	100.0	1.6	100.1	2.0	100.0	3.0	100.0	3.0	100.0	4.0	100.0	3.0

The *patina* characterisation demonstrated that both inclusions have undergone alteration processes. The relative ratio between tenorite (peak II) and cuprite (peak I) compounds on the inclusion #2 provided further insights on the burial conditions of the glass chunk. In fact, the formation of these copper oxides on the surface has been proven to be related to absence of aggressive compounds in the soil^13,14^, as well as compositional variations of metal alloys. Studies about solid-state electrochemistry of copper and bronzes artefacts showed that the relationship between cuprite and tenorite (i(II) vs. i(I)/i(II)) can help in the discrimination of corrosion compounds formed on the surface^12,15^. Figure 2Sa,b (Supplementary Materials) details the results obtained from the sampling points of the inclusion #2 mapped in Fig. 5a. Most samples showed a high percentage of PbO (2B, 3B, 3A in Fig. 2Sa,b) whereas only 4B was uniquely characterised by copper oxides. Samples 1A and 1B were composed of a mixture of these oxides.

#### Internal examination of inclusion #2

**Figure 6:**
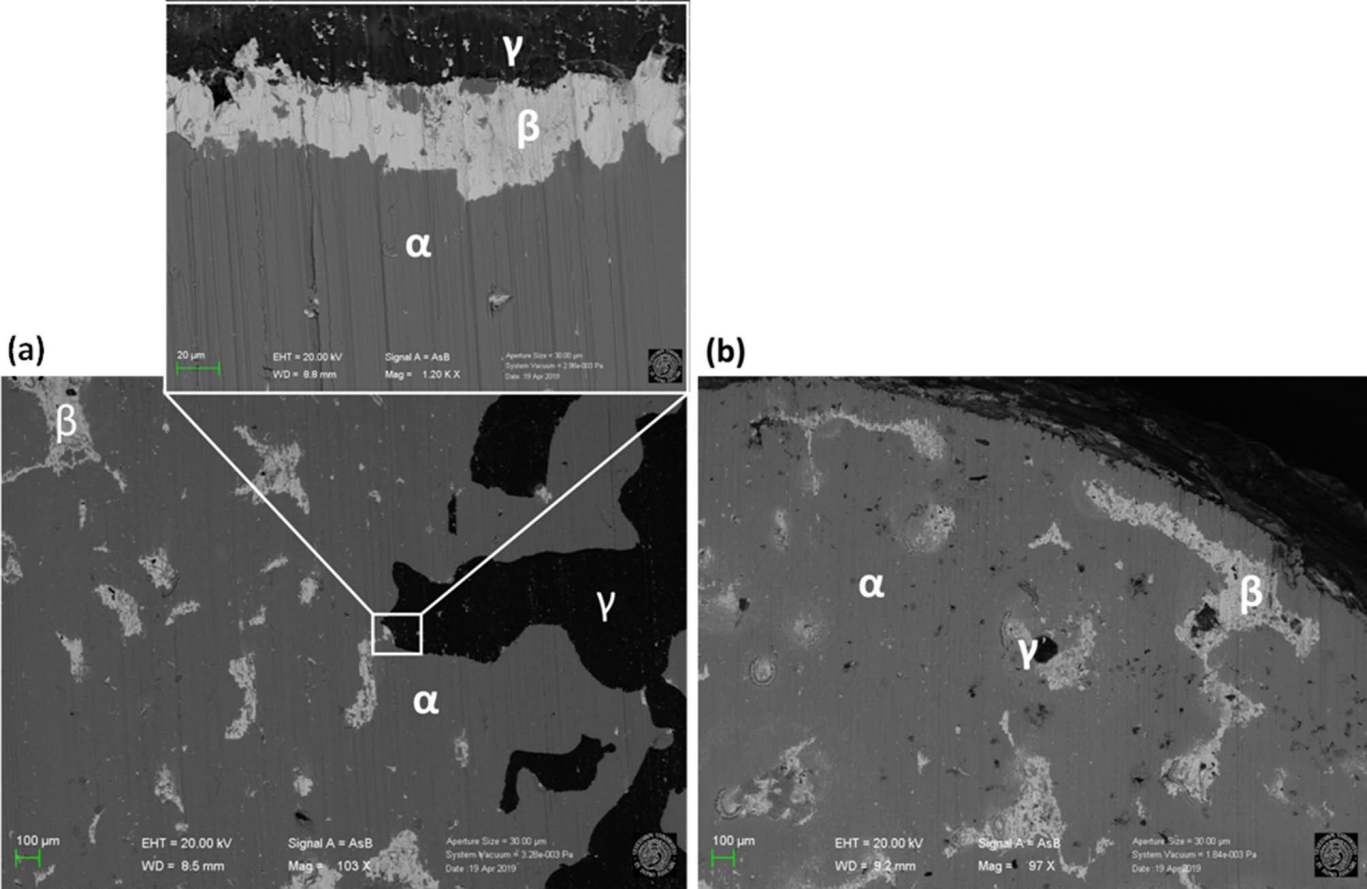
Surface characterisation by FESEM at 20 keV of the bulk matrix of inclusion #2 after sectioning: image of the interface between the three main regions observed with a magnification of 103 × and a zoom-in on the profile of the darker region (**a**). Image of the area next to the sphere perimeter with a magnification of 97 × (**b**). Inset: labels of the different phases identified, respectively the ɑ-phase (light grey) made of a copper-tin alloy, the β-phase (white) consisting of lead oxide and the γ-phase (black) made of glass. (Image: Davide Cristofori, Giulia Moro).

The partitioning of the inclusion #2 exposed a portion of its bulk material, which was analysed via FESEM-EDS: the results of this analysis are illustrated in Fig. 6. FESEM-EDS showed the presence of three different phases, indicated as α, β, γ.

The α-phase consisted of a metal matrix of a copper alloy with 85 wt% of copper and 7.6 wt% of tin (see EDS data of area 1 in Fig. 6). A heterogeneous distribution of microstructures, “elongated islands”, different in shape and size (about 100–200 μm) was observed in the α-phase. The “islands” were mainly constituted of lead (54.6 wt%) and were named β-phase. β-phase had a porous and dendritic microstructure, which is typical of lead in the complex alloys^16–18^.

The presence of lead islands within the bronze matrix can be explained considering the very low miscibility of lead in copper: when the two metals are mixed in liquid phase, copper solidifies at a higher temperature than lead, which therefore is still in the liquid phase^19,20^. The solidification of lead in a Cu–Pb system could be reached only at the alloy eutectic temperature (326 °C): this induced the formation of grain boundaries that were observed in the cross sections^16^. The presence of an unmixed phase of lead provided an additional indication of the casting condition and of the formation of the metallic object in the glass waste. In fact, lead could remain in the copper matrix forming small islands immersed in the α-phase only if the cooling rate is sufficiently fast^21,22^. On the contrary, under slow cooling rate, the two phases tend to separate, and lead is expulsed from the matrix. In addition, the β-phase had no organised structure or orientation and this indicates that during the solidification no external forces or mechanical processes were acting on it^16,23,24^.

FESEM-EDS analysis of the bulk matrix revealed also the presence of a third phase, the γ-phase in Fig. 6. The γ-phase was composed by Si, Na, Mg, Ca, Mg and K (details in Table 3) and it was confined in a macro-region well-separated from the other two phases. The composition of the γ-phase matched the main components of Roman glass material^25^. The presence of internal glass veins and their distribution in the bulk indicated that the metal inclusion was not formed by a single object melting within the fused glass, but rather it formed because of metallic agglomeration.

**Table 3:** Elemental composition of the sampling points of the bulk of inclusion #2 showed in Fig. 6. Data collected with FESEM-EDS.

Element	Z	Zone α		Zone β		Zone γ	
		wt%	σ wt%	wt%	σ wt%	wt%	σ wt%
Carbon	6	7.0	0.9	8.8	1.1	7.1	1.0
Oxygen	8	–	–	14.8	1.^-^	45	5
Sodium	11	**–**	**–**	**–**	**–**	11.8	0.7
Magnesium	12	–	–	–	–	0.79	0.07
Aluminium	13	–	–	–	–	1.49	0.09
Silicon	14	–	–	–	–	23.7	0.9
Calcium	20	–	–	–	–	4.35	0.14
Copper	29	85.4	1.8	20.5	0.5	2.65	0.10
Tin	50	7.6	0.2	1.34	0.07	–	–
Lead	82	–	–	54.6	1.5	2.62	0.13
Sum		100	2	100	3	100	5

### Metallic inclusions’ formation process

The multimodal analytic workflow applied on the two inclusions provided clear indications of their material composition and structure upon which to base hypotheses on their development process. Given the presence of glass within the metal as well as metallic fragments and separated phases, the formation of the two near-spherical inclusions was correlated to the occurrence of a colloidal system in the glass furnace induced by the dispersion of metal powder or scraps within the melted glass.

A colloid is a heterogeneous system that is made up of dispersed insoluble or soluble phases in a dispersion medium. Unlike solutions, which are formed by a single phase, colloidal suspensions are made of phase separated mixtures and these are subject to interface interactions. The physical and chemical properties of this system therefore strictly depend on its colloidal nature as the particles dispersed in the bulk usually have different properties and behaviour with respect to the continuous phase. This is the case of metal scraps or powders introduced in melted glass: at elevated temperature (around 900 °C), glass melts whereas copper and bronze are in a semi-solid phase. Notably, size, shape, composition and crystalline structure of the dispersed particles are fundamental in influencing the subsequent behaviour of the colloidal system.

The most important parameter for the stability of the colloid is the surface/mass ratio of the dispersed species. In fact, with an increasing surface/mass ratio of the dispersed particles, the system becomes thermodynamically unstable and tends spontaneously to separate from the continuous phase. The tendency of the particles to agglomerate is governed by the Van der Waals electrostatic and magnetic forces as well as the short inter-particle distances^26–28^: the metallic particles reach a more thermodynamically stable condition, while increasing their mass/size and agglomerating, as this reduces their interfacial area. It follows that without any counteractive repulsion force, the particles are naturally prone to aggregate, agglomerate or coalesce^29,30,31^. When the colloidal solution contains the maximum concentration of the dispersed phase, consistent with its solubility limit, no further dispersed phase may be added without precipitation. In conditions of supersaturation of the solution, the dispersed particles can form a single semi-rigid mass consisting of an irregular structure, which is induced to assume a spherical geometry because of the surface tension^26,32,33^.

**Figure 7:**
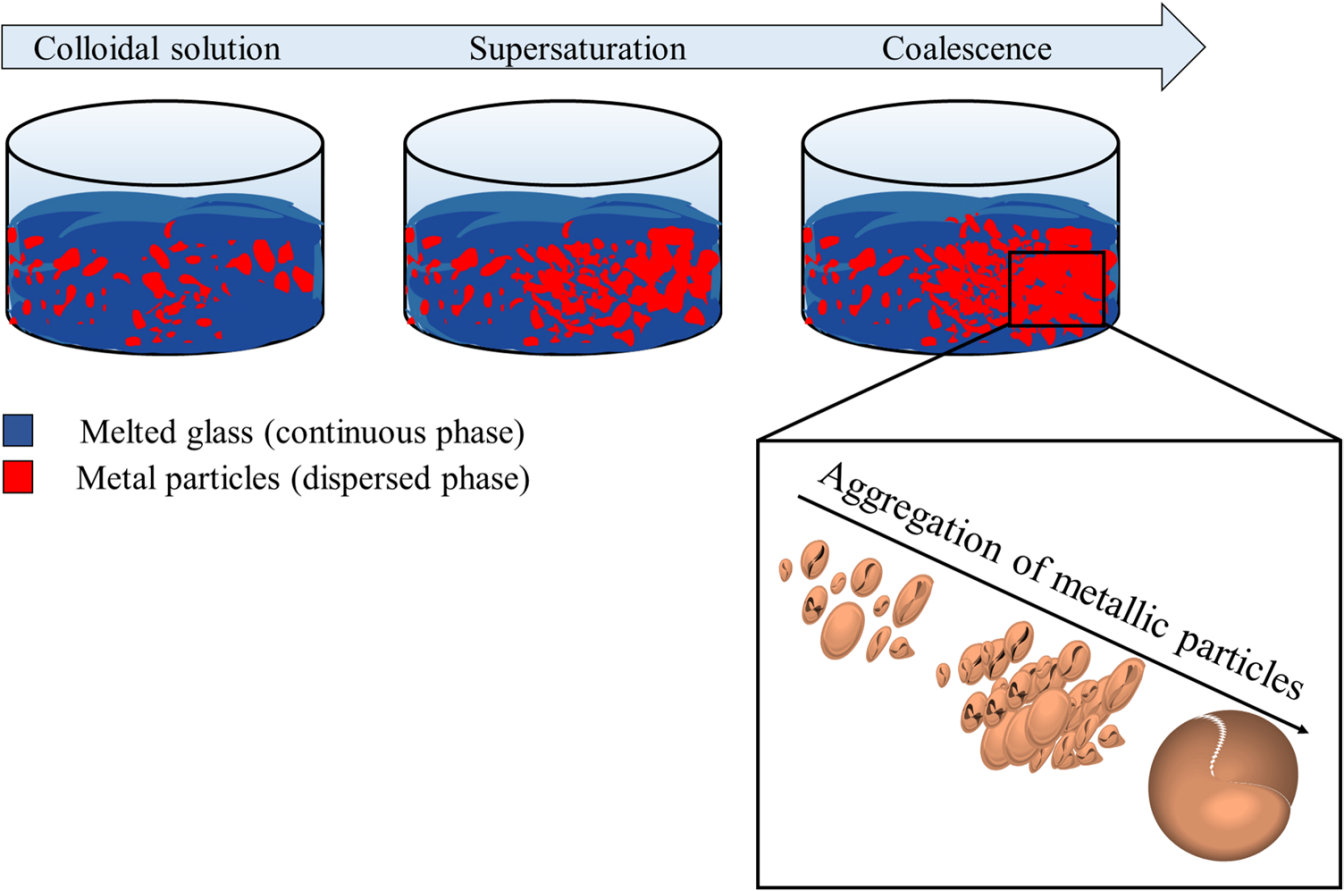
The formation of the two spheres by coalescence of metal powder used for colouring glass. (Image: Francesca Di Turo).

This scenario can explain the formation of metallic inclusions found in ancient glass slags, a phenomenon that can be related to the fundamentally experimental nature of the glass colouring approach in antiquity. The hypothesis is that during the colouring process of the glass batch, an excessive quantity of metal scraps or powder could be added to the melted glass. The metal particles would then not fully disperse in the glass and the excess of metals would precipitate because of the mechanism described above and illustrated in Fig. 7. The coalescence process would then be favoured by the high temperature and it would be followed by an increase in the viscosity of the system; the final aggregation of the particles would result in a spherical structure that incorporates the continuous phase (the glass) during the formation. A similar event has likely generated the inclusions found in the Aquileian glass slags, where an overdose of metal components (Cu or combined Pb, Cu and Sn) was probably added to the melting glass to produce a colour variation, ending up in a supersaturation of the glass-metal colloidal system that led to the aggregation and sedimentation of the metal inclusions. The phenomenon of surface tension might have ultimately determined the near spherical shape of the solid object.

While the process triggering the development of the inclusions can be considered substantially the same, the differences in the dimension of the metallic fragments inside the inclusions highlighted by the CT scanning could be explained by considering some parameters that can play a role in the process. First, the materials forming the inclusions were different (inclusion #1 was made uniquely of copper, while inclusion #2 was made of leaded bronze): this could have implied a different behaviour of the colloid at elevated temperatures. Secondly, the glass-working process might have been conducted at different temperatures and different reducing/oxidising conditions in the furnace. Last, the different size of the two forming objects might have affected the solidification temperature and consequently the internal morphology and distribution of the mixed phases.

## Discussion

The results of this study have indicated that the formation of the two metallic inclusions could be related to a fortuitous event associated with the colouring process of glass. This assumption is substantiated by the fact that they were mainly composed of copper (inclusion #1) and leaded bronze (inclusion #2), two alloys/metals known to be used in the past as colouring agents, and further corroborated by other aspects, such as the shape of the items and their internal structure, which can be related to their origination process. Both inclusions had in fact a near-spherical shape, which can be linked to a surface tension process, and an internal morphology (at least as far as inclusion #1) showing an uneven core composed by metallic fragments separated by branched veins of glass, indicating that the internal bulk was composed by heterogeneously dispersed mixing phases (specifically, bronze, lead and glass). This led to conjecture that the two metallic inclusions got formed following an aggregation process induced by a supersaturation condition reached by the metal–glass system. What occurred in this case was that copper or bronze were introduced in the crucible in the form of scraps or powder and got dispersed in melted glass. While in the crucible at high temperatures (estimated over 900 °C), the melted glass behave as a continuous phase and the metal particles as the dispersed one. Given the excess of metallic powder, the latter were no more soluble, thus precipitating and forming a new body.

The findings of this study play a critical role in substantiating some archaeological hypotheses. The presence of these by-products, which are correlated with the working of glass, corroborates the suggestion that near the premises of the chunks’ discovery could have existed a secondary glass-working site. Beside the very high number of chunks identified (450 + collected, but many more spotted on site and not recovered), which might be—by itself—a clear indication of a glass workshop in those grounds, the discovery of crucible and refractory’s fragments, glass drops and cullets found in association with the glass slags can be considered a further signal of such presence. To date, archaeological evidence of glass furnaces in the Aquileian area has not yet been found, although hinted by ancient written sources: the two metallic inclusions (and a few similar others of smaller diameter ones, observed—but not yet studied—embedded within other glass chunks from the same assemblage) together with the associated glass-processing’s residuals could further strengthen the hypothesis of a flourishing industry of glass working in the suburban area of ancient Aquileia.

## Methods

### X-ray computed tomography

The investigations were performed with a laboratory-based micro-tomographic facility designed for high-resolution investigation of objects. The micro-CT system can range voxel size by changing the magnification factor (ratio between source-detector and source-rotation centre distances) from about 5 to 20 microns. A customizable choice of scanning parameters enables to enhance the image quality depending on the sample characteristics (especially size and density). Small specimens can be scanned with high accuracy, while for bigger samples the field of view can be extended by means of mechanical translation of the detector (tile scanning techniques)^34,35^. The system comprises a micro-focus X-ray tube (Kevex mod. PXS10, max. voltage of 130 kV and 7–100 µm focal spot), a high-resolution CCD X-rays detector (Photonic Science VHR camera with a GOS scintillator, 2004 × 1336 pixels, 3.6 × 2.4 cm^2^ FOV) and a Physics Instrument precision rotator, mounted on the top of a tip-tilt alignment stage. The system works in cone-beam geometry with a synchronous step by step scanning routine that includes frame averaging to increase signal to noise ratio. Parameter settings for scanning the metallic inclusions were: (tube) 130 kVp, 50 μA and 0.1 mm Pb filtration; (camera) 3.5 s exposure time with 8 frames average for inclusion #1 and 16 for inclusion #2; (distances) source-object distance (SOD) of 112.5 mm and of a source-detector distance (SDD) of 234.5 mm, voxel size of 8.64 μm; (mechanics) scanning over 180° with 900 projections. The reconstructions were performed with in-house developed software based on the Feldkamp, Davis, and Kress (FDK) algorithm^36,37^.

Additional processing—the *beam-hardening* correction—was required to optimise the reconstruction due to the concurrence of highly absorbing materials and a relatively low energy used for the investigations. This was implemented following the procedure illustrated by Zhao et al.^38^.

The 3D rendering and the image display were done with 3D Slicer (an open- source software), VGStudio Max (by Volume Graphics Inc., USA) and ImageJ (an open- source software). VGStudio Max and ImageJ were also used to perform the histogram plots analysis and the volumetric fraction evaluation of the inclusions.

### Voltammetry of Immobilized microparticles

All data were collected at room temperature (298 K) under nitrogen atmosphere using a CH200A Potentiostat (Cambria Scientific, Llwynhendy, Llanelli UK). The electrochemical set-up used was composed by a three-electrode cell with glass carbon working electrodes (GCE), a KCl-saturated Ag/AgCl reference electrode and a platinum wire counter electrode. All the potential reported in the text are referred to the KCl-saturated Ag/AgCl that has a standard potential of − 45 mV vs SCE. Acetate buffer pH 4.75 was used as supporting electrolyte. Sample-modified electrodes were prepared by immobilising microparticles with Nafion. Before immobilising sample micro-particles, the GCE were polished with alumina slurries (three particle sizes: 1, 0.3, 0.05 µm) and their performances were tested by CV. The polished GCE were finally dried under nitrogen flux. The stock solution of Nafion® 0.5% w/v was prepared by 1:10 dilution with methanol of 5% Nafion® solution. After the micro-particle sampling, the scratched powders were deposited directly at the GCE surface, immobilized by adding 2–3 μL of Nafion® 0.5% solution and let dry at room temperature, as previously reported in^39^. Square wave voltammetry was used as a detection method using the following parameters: potential window from − 1.0 to + 1.0 V, scanned in the negative (cathodic) or positive (anodic) direction; amplitude 0.025 V; frequency 5 Hz; potential step increment 0.004 V.

### Field emission scanning electron microscopy with energy dispersive spectroscopy (FESEM-EDS)

The analyses were carried out with a FESEM Zeiss Sigma|VP operated at 20 kV and equipped with an energy-dispersive X-ray spectrometer (EDS) Bruker Quantax 200, a system of the silicon drift detector (SDD) type with a 30 mm^2^ collection window.

## Supplementary Information

Supplementary Information.
