# Supplementary material for: Chemical analysis and computed tomography of metallic inclusions in Roman glass to unveil ancient coloring methods
Source: Sci Rep. 2021 May 27;11:11187. doi: 10.1038/s41598-021-90541-8 (PMC8160351; doi:10.1038/s41598-021-90541-8)
Supplement: Supplementary file 1 — Supplementary Information. [file 41598_2021_90541_MOESM1_ESM.pdf]

Supplementary Materials

Chemical analysis and computed tomography of metallic inclusions in Roman glass to unveil ancient coloring methods

Francesca Di Turo[1], Giulia Moro[2], Alessia Artesani[1], Fauzia Albertin[3,4,5], Matteo Bettuzzi[4,5], Davide Cristofori[2,6], Ligia Maria Moretto[1,2], and Arianna Traviglia[1,*]

[1] Istituto Italiano di Tecnologia, Center for Cultural Heritage Technology (CCHT), Venezia, 30175, Italy
[2] Ca' Foscari University of Venice, Department of Molecular Science and Nanosystems, Venezia, 30172, Italy
[3] Historical Museum of Physics and the Enrico Fermi Study and Research Center - CREF, Roma, 00184, Italy
[4] University of Bologna, Department of Physics and Astronomy, Bologna, 40127, Italy
[5] INFN - National Institute of Nuclear Physics, Bologna, 40127, Italy
[6] Ca' Foscari University of Venice, Centre for Electron Microscopy "Giovanni Stevanato", Venezia, 30172, Italy

[*] arianna.traviglia@iit.it

Sectioning procedure for inclusion #2

The sectioning of this archaeological item was undertaken using a diamond wheel saw, with diamond grains in the 1-3 µm size range. The inclusion #2 was clamped in the sample holder prior to the cut (see Fig.1Sa). The wheel was set to rotate at low speed for minimising the pressure on the sample and reducing damages induced by the abrasion process. The object was sectioned along a plane set slightly off the central axe of the near-spherical item, as showed in Fig.1Sb.

Once sectioned, inclusion #2 was carefully cleaned with a brush to remove powder and dust residues.

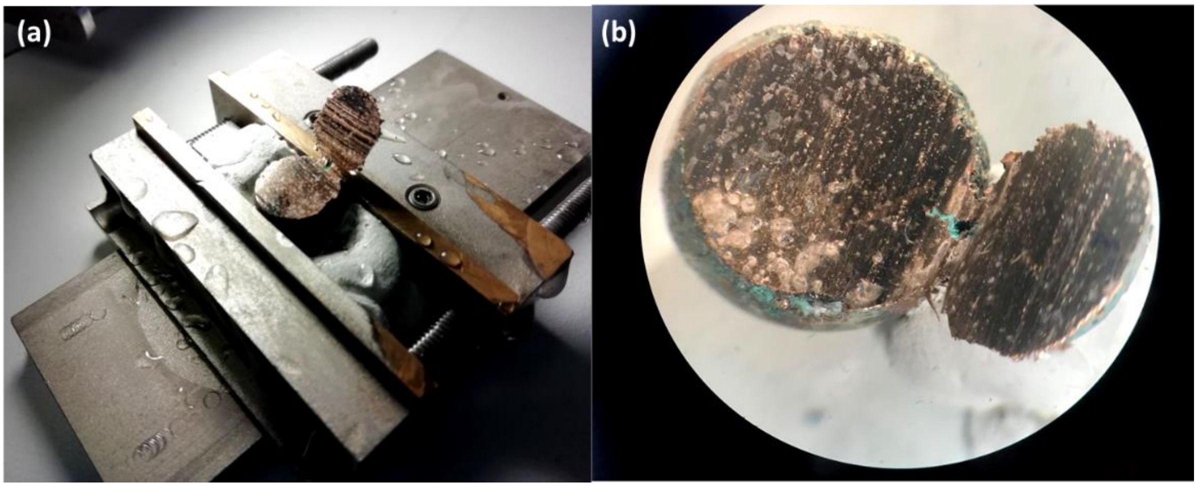

Figure 1S - (**a**) inclusion #2 blocked in the saw sample holder after sectioning and (**b**) inclusion #2 sectioned.

VIMP analysis

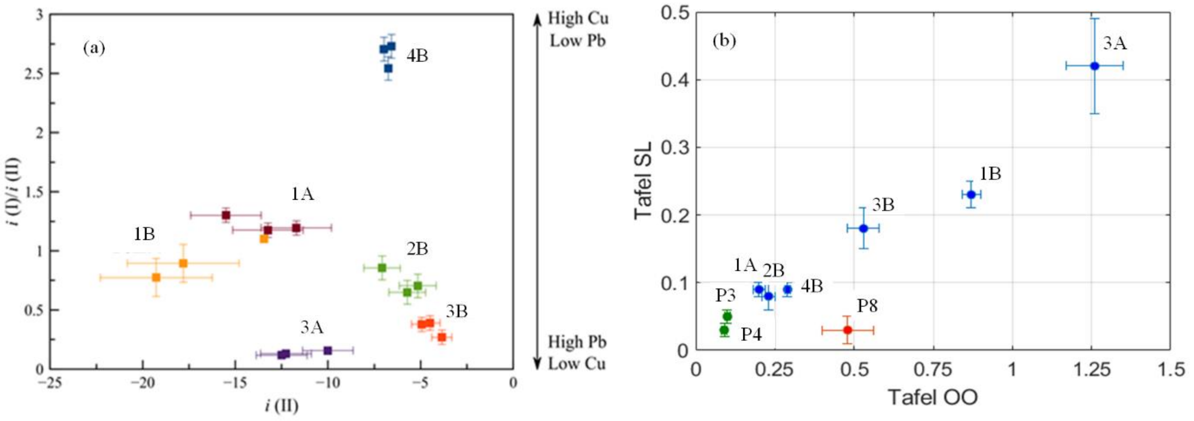

Figure 2S. (a) Variation of *i*(II) vs. *i*(I)/*i*(II) for the different areas sampled. Differences can be detected when Cu is higher than Pb or *vice versa*. **(b)** Diagram obtained by the modified Tafel analysis on the copper-based compounds of the samples. Two-dimensional plot shows the slope and the ordinate at the origin, calculated according to the procedure proposed by Domènech-Carbò et al.[Doménech-Carbò, A. et al. Electroanalysis 23, 2803–2812 (2011)][Doménech-Carbò, A. et al. Anal. Chim. Acta 680, 1–9 (2010)]. The sampling point 2A was excluded because can be assigned to lead oxide.

Tafel analysis enabled to identify copper-based corrosion products thanks to the electrochemical response of the rising portion of the voltammetric curves. The results in Fig. 2Sb showed a heterogeneous distribution of the sampled points. The data coming from inclusion #2 fall in the low region of the diagram (2B, 1A) indicating the presence of cuprite. The distribution in the Tafel graph of points 3A and P8 was associated to the presence of different compounds in these areas, like Al, Mg and Fe. P8 was sampled in the bulk of inclusion #2 after sectioning therefore it is not indicated in Fig. 5a. 3A was sampled in a region, reach of Pb, and its voltammetric signal was strongly influenced. P3 and P4 are sampled points of inclusion #1 and are presented here for comparison.
